# Combatting Antibiotic Resistance Using Supramolecular Assemblies

**DOI:** 10.3390/ph15070804

**Published:** 2022-06-28

**Authors:** Shuwen Guo, Yuling He, Yuanyuan Zhu, Yanli Tang, Bingran Yu

**Affiliations:** 1 Key Laboratory of Analytical Chemistry for Life Science of Shaanxi Province, School of Chemistry and Chemical Engineering, Shaanxi Normal University, Xi’an 710100, China; zyyzyy304@snnu.edu.cn; 2 Institute of Basic and Translational Medicine, Xi’an Medical University, No. 1 Xinwang Road, Xi’an 710021, China; heyuling0925@163.com; 3 State Key Laboratory of Chemical Resource Engineering, Key Lab of Biomedical Materials of Natural Macromolecules (Beijing University of Chemical Technology, Ministry of Education), Beijing 100029, China

**Keywords:** supramolecular assembly, antibacteria, antibiotic resistance

## Abstract

Antibiotic resistance has posed a great threat to human health. The emergence of antibiotic resistance has always outpaced the development of new antibiotics, and the investment in the development of new antibiotics is diminishing. Supramolecular self-assembly of the conventional antibacterial agents has been proved to be a promising and versatile strategy to tackle the serious problem of antibiotic resistance. In this review, the recent development of antibacterial agents based on supramolecular self-assembly strategies will be introduced.

## 1. Introduction

Antibiotic resistance is a growing problem that causes 700,000 deaths per year worldwide, and a recent prediction indicates that bacterial infections will cause 10 million deaths by 2050 [1,2,3,4,5,6]. The rapidly growing antibiotic resistance is mainly driven by misuse of antibiotics in human medicine and abuse of antibiotics in animal medicine and husbandry [7,8,9,10]. The emergence of multi-drug-resistant infections makes the situation worse, as multi-drug-resistant infections require prolonged and high-cost antibiotic therapy [11,12,13,14]. However, the investment in research and development of new antibiotics is diminishing, as the emergence of resistance has outpaced the development of new antibiotics [15,16,17,18]. Therefore, developing novel approaches to tackle the serious antibiotic resistance crisis is urgently needed.

Researchers in relevant fields are making great efforts to discover new drugs and develop new antibacterial strategies and technologies to address the serious issues caused by antibiotic resistance [19,20,21,22,23,24,25,26]. Supramolecular self-assembly focuses on the autonomous organization of components into multi-component systems through intermolecular noncovalent interactions, including electrostatic, hydrophobic, hydrogen-bonding, metal-ligand coordination, charge–transfer, van der Waals, and π-π stacking interactions [27,28,29,30,31,32]. The dynamic properties and integration features of supramolecular self-assembly endow the assembly with extraordinary functions, which cannot be empowered by traditional covalent modification strategies. Therefore, supramolecular self-assembly strategies have demonstrated great potential applications in the biomaterial field. In particular, supramolecular self-assembly strategies have been applied in combating bacteria and bacterial biofilms.

In this review, we briefly highlight and discuss the recent development of antibacterial agents based on the supramolecular self-assembly strategies, which involve the above-mentioned noncovalent interactions. The typical and novel supramolecular self-assembly strategies, which could improve the therapeutic performance of antibacterial agents and overcome their shortcomings, will be focused on in this review. The review is divided into six sections according to the type of conventional antibacterial agents constituting the supramolecular self-assembly, including antibiotics, antimicrobial peptides, cationic surfactants and polymers, antibacterial photodynamic therapy (aPDT) agents, antibacterial photothermal therapy (aPTT) agents, and macrophages.

## 2. Supramolecular Self-Assembly of Antibiotics

Since their introduction in the 1940s, antibiotics have been heavily applied to treat infectious diseases. After the “Golden Age” of antibiotics, semisynthetic and chemically modified antibiotics have been deemed as the bright way to develop new antibiotics [33,34,35,36,37]. However, these innovative compounds cannot address the antibiotic-resistant issues as bacteria could rapidly acquire tolerance after a period of gene response. Thus, developing a novel antibacterial strategy based on the existing antibiotics to combat resistance is greatly needed. Supramolecular self-assembly of antibiotics is a promising strategy to improve therapeutic efficiency, reduce nonspecific cytotoxicity, and depress drug resistance to antibiotics. Bhosale and coworkers have summarized the supramolecular self-assembly of amino-glycoside antibiotics [38], and for readers interested in this aspect, please refer to the review.

**Figure 1:**
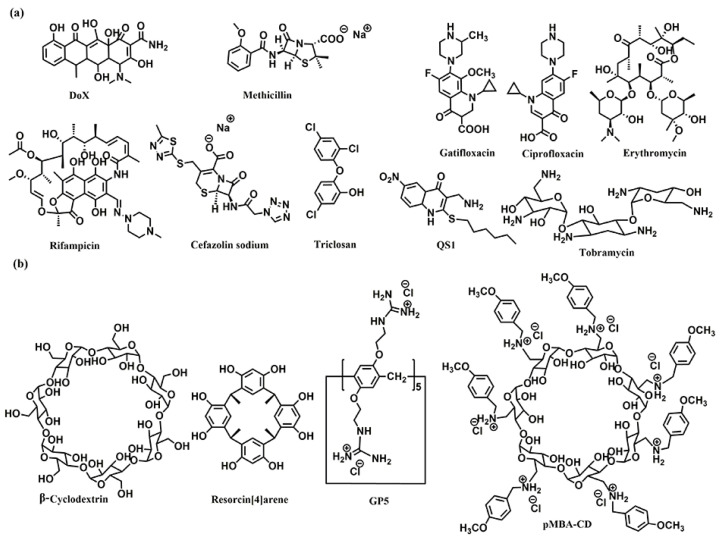
Chemical structures of (**a**) the antibiotics, and (**b**) the co-assembly materials used for fabricating supramolecular self-assembly of antibiotics.

The host–guest complexations between antibiotics and macrocyclic hosts are highly potential approaches to enhance antibiotics activity. Sinisterra et al. reported that the complexation of doxycycline (Dox) and β-cyclodextrin (β-CD) showed greater antimicrobial activity than free doxycycline (Figure 1) [39]. Isothermal titration calorimetry (ITC) data indicated that the free doxycycline and the Dox/β-CD complexation bind with the cell membrane through different interactions. The interaction of free doxycycline with the cell membrane is an ion-paring and hydrogen bond, while the interaction of Dox/β-CD complexation with the cell membrane is a much stronger hydrogen bond. The Dox/β-CD complexation could serve as a local and sustained source to improve treatment efficacy. Dend et al. also reported the complexation of per-6(4-methoxylbenzyl)-amino-6-deoxy-β-cyclodextrin (pMBA-βCD) and methicillin (Met) to increase the water solubility of Met and its antibacterial activities against methicillin-resistant Staphylococcus aureus (MRSA) [40], which was presumed that the Met/pMBA-βCD complex improved the affinity between the active β-lactam moiety and the narrow active site groove of MRSA PBP2a, and facilitate the acylation [41,42] (Figure 1). The solubility and antibacterial activity of ciprofloxacin were also enhanced by Mono-6-deoxy-6-aminoethylamino-β-cyclodextrin (mET-βCD, Figure 1) [43]. NOESY NMR indicated that the ethylenediamine moiety of mET-βCD induces stable hydrogen bonding with primary hydroxyls of β CD, leading to the formation of this oval-shaped cavity, which could encapsulate quinolone and the cyclopropyl groups of ciprofloxacin. D-mannose and D-glucose-grafted cyclodextrins were also developed as “Trojan Horse”-like nanocarriers for loading hydrophobic antibiotics (ciprofloxacin, erythromycin, and rifampicin) and potentiate their activity, as the functionalized sugars on CD are chemoattractant for the bacteria and could promote uptake (Figure 1) [44].

Kumari et al. also fabricated the host–guest complexation between resorcin[4]arene and gatifloxacin to improve antibacterial activity [45]. These host–guest complexations indicated that strategies based on host–guest interactions are highly potential approaches to constructing novel antimicrobial formulations, which would improve antibacterial activity, water-solubility, and biocompatibility of traditional antibiotics.

**Figure 2:**
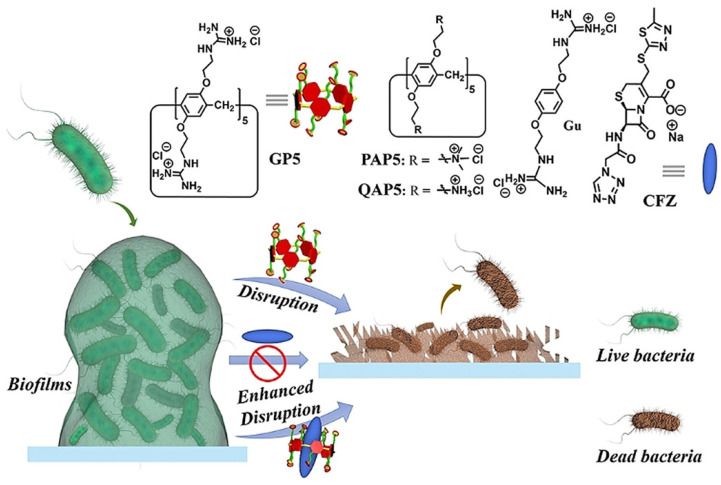
Disruption of biofilms by GP5 and GP5⊃CFZ. Reprinted with permission from Ref. [48]. Copyright 2021 Wiley-VCH.

Microbial biofilms showed restricted drug accessibility and antibiotic resistance, and most cells in the biofilms changed their metabolic mode to a dormant state. All of these factors result in great challenges in disrupting biofilms [46,47]. Supramolecular self-assembly of antibiotics is also a potential strategy to disrupt biofilms. Wang and coworkers also developed host–guest complexation between guanidinium per-functionalized pillar[5]arene (GP5) and the antibiotic cefazolin sodium to synergistically eradicate *Escherichia coli* (*E. coli*) biofilm [48]. In vitro experiment showed that GP5 could disrupt biofilm, as guanidinium per-functionalized pillar[5]arene could penetrate through biofilm barriers and destroy biofilm-enclosed bacteria. After GP5 formed the host–guest complex with cefazolin sodium, the host–guest complex penetrated through biofilm due to the high penetrability of preorganized multiple guanidiniums on pillar[5]arene skeleton (Figure 2). The GP5 and cefazolin sodium, therefore, could effectively breach the bacterial membranes and work synergistically to kill the bacteria within the biofilm matrix.

Encapsulation and delivery of antibiotics through the self-assembly of lipids is a promising way to enhance the bioavailability of antibiotics [49]. Macrophage membrane encapsulated antibiotic was construed to selectively enter into the infected macrophages and efficiently kill intracellular bacteria [50]. The hydrophobic triclosan and hydrophilic ciprofloxacin were covalently conjugated together, and the obtained amphiphilic prodrug self-assembled into nanoparticles. With further encapsulation with macrophage membranes, the nanoparticles showed similar Toll-like receptor expression and negative surface charge as their precursor murine macrophage/human monocyte cell lines. Such features allowed uptake of the infected macrophages/monocytes through positively charged, lysozyme-rich membrane scars created during staphylococcal engulfment.

Combined antibiotic administration based on co-assembly is also an effective way to eliminate resistant infections. Lehr et al. developed a co-assembled nanoparticle of synthesized amphiphilic lipid (squalenyl hydrogen sulfate), hydrophilic antibiotic tobramycin, and a quorum sensing inhibitor (QS1) to synergistically eradicate *Pseudomonas aeruginosa* *(P. aeruginosa*) biofilm [51].

**Figure 3:**
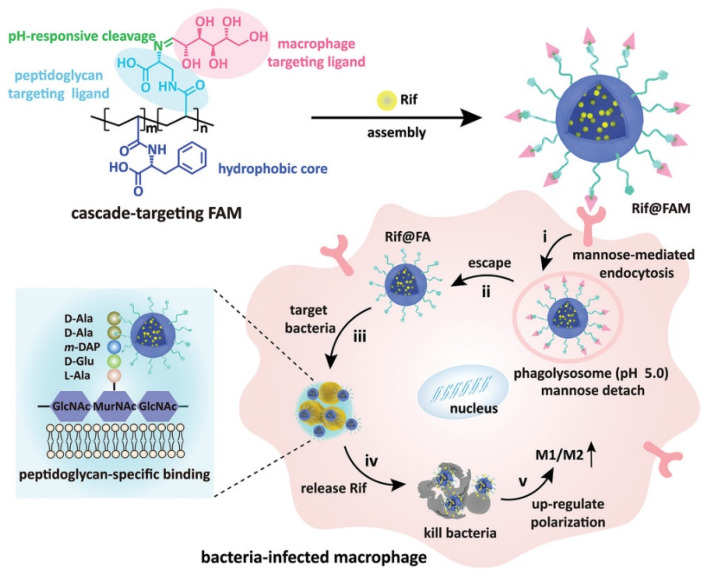
The cascade-targeting DDS eliminates intracellular MRSA. Reprinted with permission from Ref. [52]. Copyright 2022 Wiley-VCH.

Wang and coworkers fabricated cascade-targeting poly(amino acid) nanoparticles to sequentially target macrophages and intracellular bacteria and fulfill on-site antibiotic delivery. The mannose-decorated poly(α-*N*-acryloylphenylalanine)-block-poly(β-*N*-acryloyl-d-aminoalanine) could self-assemble into nanoparticles and efficiently load rifampicin due to abundant noncovalent interactions between the drug and polymer backbone (Figure 3). The mannose groups from the surface of the nanoparticles promote macrophage-specific uptake and intracellular accumulation. After exposing the D-aminoalanine moieties in the acidic phagolysosome, the nanoparticles escape from lysosomes and target intracellular bacteria through peptidoglycan-specific binding. Subsequently, rifampicin was precisely released regardless of the states and locations of the intracellular MRSA [52].

## 3. Supramolecular Self-Assembly of AMPs

Antimicrobial peptides (AMPs), a group of functional peptides that display antimicrobial activities, are potential therapeutic agents due to their broad-spectrum antibacterial activities and low degrees of antimicrobial resistance [53,54,55]. As peptides are outstanding and feasible building blocks for self-assembly, the artificial peptides with self-assembling capacities have been applied in antibiotic delivery, antibacterial surface defense, bio-sensing of bacteria, and as bacteriostatic agents [56,57,58]. The self-assembly of AMPs could not only enhance antimicrobial efficacy by locally enhancing the antimicrobial peptide concentration on the bacterial surface but also promote the biological stability of the fragile peptide.

AMPs are drawing great attention in modern medicine due to their high biological activity. Their low stability leads to undesirable bioavailability, as these agents could be degraded by proteases. Further, systemic toxicity is also a major issue for some classes of AMPs. Recently, Li et al. exploited the host–guest complexion between and pexiganan (PXG) to decrease hemolysis and improve the stability of PXG [59]. The two large-sized macrocycles showed strong interactions towards PXG, giving association constants in the magnitude of 10^4^ and 10^5^ M^−1^. The host–guest complexation remarkably improves metabolic stability under endogenous proteases conditions and decreases hemolysis of PXG.

**Figure 4:**
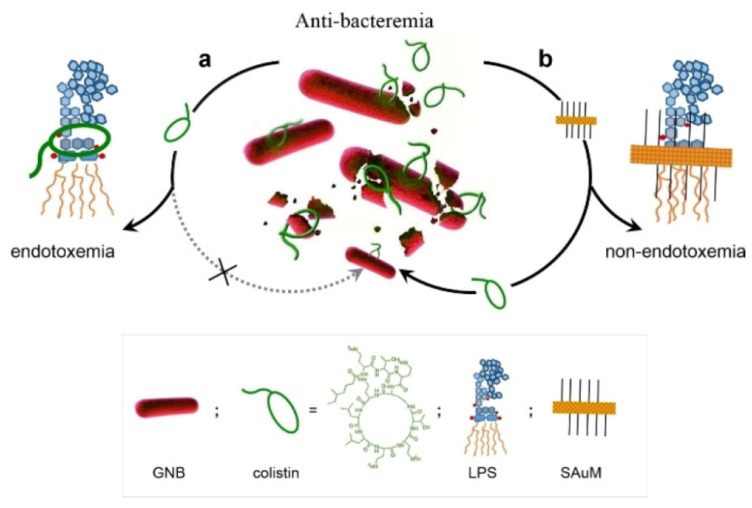
A Supramolecular Trap to Increase the Antibacterial Activity of Colistin. In Path a, free LPS can block the function of colistin. And the free LPS can be sequestered by SAuM in the circulating blood (in Path b), thus promoting the killing efficiency of colistin against GNB while also minimizing endotoxemia. Reprinted with permission from Ref. [60]. Copyright 2020, Wiley-VCH.

Lin et al. developed a supramolecular trap to boost the low-dose antibacterial activity of colistin against *E. coli* by preventing the interaction between colistin and free LPS [60]. The molecular trap was fabricated from a subnanometer gold nanosheet with methyl motifs (SAuM) and could directly target and capture free LPS by binding to and compressing the packing density of lipid A, thus reducing the risk of endotoxin-induced sepsis and preventing its interaction with colistin (Figure 4). The binding specificity assay results showed that SAuM is a strong competitor of colistin for binding with free LPS. The higher driving force of SAuM to bind with LPS is mainly due to the fact that the sheet-like structure of SAuM can precisely bind the lipid A of LPS to form a steric wall that efficiently inhibits colistin binding. This supramolecular trap allows the therapeutic window of colistin to be expanded to low-dose concentrations for the treatment of Gram-negative bacteria infections while also minimizing the risk of endotoxemia.

Tang and coworkers developed a novel peptide-based co-assembled hydrogel to effectively and specifically capture and kill MRSA bacteria. The supramolecular self-assembly of 9-fluorenylmethyloxycarbonyl-L-phenylalanin (Fmoc-L-Phe) and amino-acid-modified conjugated oligomer OTE-D-Phe formed a new and biocompatible low-molecular weight hydrogel [61]. The hydrogel was composed of a thick and rough fibrous network, which could spontaneously capture MRSA and *E. coli* efficiently. The hydrogel exhibited efficient and specific antibacterial activity against MRSA due to specific interaction with lipid domains of the MRSA membrane.

## 4. Supramolecular Self-Assembly of Cationic Surfactants and Polymers

Cationic surfactants have been extensively applied as antimicrobial agents in various fields due to their excellent bactericidal activities [62,63,64]. The cationic surfactants exert biological activity mainly by disrupting bacterial membranes via electrostatic and hydrophobic interaction, which further leads to cell lysis and bacterial death [65,66]. The cationic surfactants were once supposed to be impervious to resistance. However, after huge accumulation in the environment over the past decades, bacteria have acquired surfactant resistance [67,68,69]. Moreover, it is a great challenge to design highly antibacterial surfactants with high selectivity, as the essential factors for antibacterial activity also predominate their cytotoxicity. Recently, the application of the supramolecular self-assembly strategy for cationic surfactants has shown encouraging results in improving their antimicrobial activity, bacterial resistance, selectivity, etc.

Wang and coworkers developed supramolecular antimicrobial agents with switchable activities, which could reduce the pressure on bacteria and prevent the emergence of bacterial resistance. The supramolecular antibacterial switches were constructed based on the host–guest complexation of cucurbit(7)uril (CB[7]) and cationic surfactants (DDBAC, DDBAB, BCDAC, and DTAC) [70]. The antimicrobial ability was switched off when the alkyl chain of surfactants was encapsulated by CB[7], which concealed the active sites. The antimicrobial ability was switched on when the active sites recovered upon the addition of competitive guest amantadine hydrochloride (AD). As the exposure frequency of bacteria to active surfactants could be reduced by regulating the antimicrobial switch, the occurrence of drug resistance was prevented by the antimicrobial switch.

Wang et al. also fabricated the host–guest complexation of biocompatible CDs and cationic surfactant (tri(dodecyldimethylammonioacetoxy)-diethyltriamine trichloride, DTAD) to improve the mildness of cationic surfactant [71]. The outstanding antimicrobial DTAD showed a strong skin irritation effect due to its larger numbers of cationic charges and multiple hydrophobic chains. The host–guest complexation of DTAD and three types of CDs (α-, β-, γ-CD) could self-assemble to nanostructure with different morphology, and the antibacterial activity and mildness of DTAT were all improved by the host–guest complexation and further self-assembly.

Tang and coworkers fabricated cationic nanoparticles through the co-assembly of cationic surfactant cetyltrimethylammonium bromide (CTAB) and conjugated polymers (PFVBT), which could enhance the antibacterial effect and lower the cytotoxicity of CTAB [72]. When mixing CTAB and PFVBT together, the hydrophobic chain of the polymers was entwined with the hydrophobic part of CTAB to form the nucleus, and the hydrophilic quaternary ammonium groups formed shells on the surface, resulting in the spherical cationic nanoparticles. Instead of light source requirement, which is vital for the antibacterial activity of most conjugated polymers and nanoparticles, the cationic nanoparticles showed efficient and broad-spectrum biocidal activities in the dark. This work provides a new way to combine cationic surfactant and conjugated polymers together to combat bacteria.

**Figure 5:**
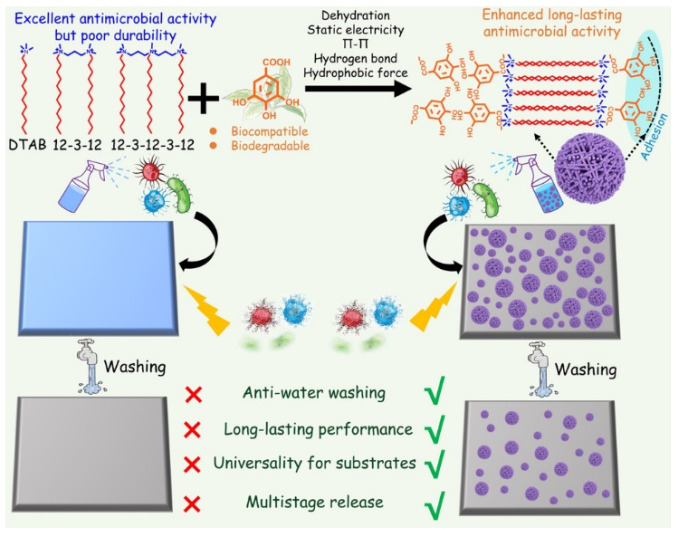
Construction of substrate-adhesive aggregates (hexagonal columns interpenetrated spheres, HCISs) based on noncovalent assembly of gallic acid (GA) and three quaternary ammonium surfactants (QASs). Reprinted with permission from Ref. [73]. Copyright 2021, Wiley-VCH.

The commonly used antibacterial quaternary ammonium surfactants (QAS) generally lack long-lasting performances. The frequent usage of these agent cause accumulation in the environment, which would exert a huge burden on the ecosystem and selective pressure on bacteria, triggering the emergence of antimicrobial resistance. Supramolecular self-assembly is a potential strategy to fabricate long-lasting antimicrobial agents. For example, Wang et al. fabricated long-lasting antimicrobial aggregate by co-assembling QAS and biocompatible gallic acid through multiple synergistic noncovalent interactions, including electrostatic interaction, hydrogen bonding, hydrophobic interaction, and π-π stacking [73]. Three QASs, including dodecyltrimethylammonium bromide (DTAB), trimethylene-1,3-bis-(dodecyldimethyl-ammonium bromide) and methyldodecylbis[3-(dimethyldodecylammonio) propane] ammonium tribromide were chosen to co-assemble with gallic acid (GA) (Figure 5). GA could counterbalance the charge repulsion of the surfactant headgroups after electrostatic binding with the surfactant, and GA could serve as a linker to adhere surfactant micelles together through intermolecular hydrogen bonding of the phenolic hydroxyl groups. The parallelly stacked micelles formed hexagonal columns, and the hexagonal columns interpenetrated spheres (HCISs) were finally generated when these hexagonal columns intersperse at different orientations. The strong dehydration of HCISs and their adhesion force to substrates endowed the HCISs with anti-water washing properties and long-lasting antimicrobial performance.

Traditional antibiotic abuse has caused a lot of health problems, including antimicrobial resistance. In order to tackle these problems, Lei and coworkers investigated two self-assembling modes between berberine (BBR) and flavonoid glycosides and compared their antibacterial potency (Figure 1). Based on electrostatic and hydrophobic interactions, the mixture of BBR and baicalin formed nanoparticles in an aqueous solution, while BBR and wogonoside generated nanofibers [74]. The nanoparticles exhibited significantly enhanced bacteriostatic activity, whereas nanofibers showed a weaker effect than BBR. The enhanced bacteriostatic activity of nanoparticles is likely attributed to their stronger affinity to bacteria as hydrophilic glucuronic acid was distributed on the surface of nanoparticles. Using a similar strategy, they also developed nanoparticles by the self-assembly of BBR and cinnamic acid to combat MRSA and biofilm [75]. The self-assembly of the nanoparticles was governed by hydrogen bonds and π-π stacking interactions.

A precise pesticide delivery nanoplatform was fabricated by iron mineralization after electrostatic self-assembly between sodium lignosulphonate (SL) and dodecyl dimethyl benzyl ammonium chloride (DDBAC). The nanoplatform was a core–shell structure, which was excellently stabilized by iron mineralization. The outer layer of the shell was constructed by in situ mineralization with iron ions, while the endothecium of the shell was formed by the electrostatic self-assembly of SL and DDBAC. The nanoplatform was suitable for encapsulating agrochemicals with hydrophobicity and low volatility. The nanoplatform showed a specific response to alkalescency and laccase in the alimentary tract of insects and also improved the photostability and environmental security of agricultural chemicals [76].

Shen et al. designed an electrochemical redox-controlled bacterial inhibition agent [(12-ferrocenyl) benzalkonium bromide (FBZK)] containing ferrocene quaternary ammonium salt based on supramolecular self-assembly and disassembly [77]. They demonstrated that using a redox-active cationic antibiotic to regulate antibacterial activity with an alternating electric field takes the critical micelle concentration (CMC) as the boundary, inhibition of antimicrobial activity by redox-induced changes in hydrophilicity and hydrophobicity. At a concentration over the CMC, self-assembly into micelles led to a decrease in antibacterial activity; on the contrary, the disassembly of micelles can enhance the bactericidal effect. When the concentration was below the CMC, REFBZK with a hydrophobic tail penetrated more easily into the membrane.

**Figure 6:**
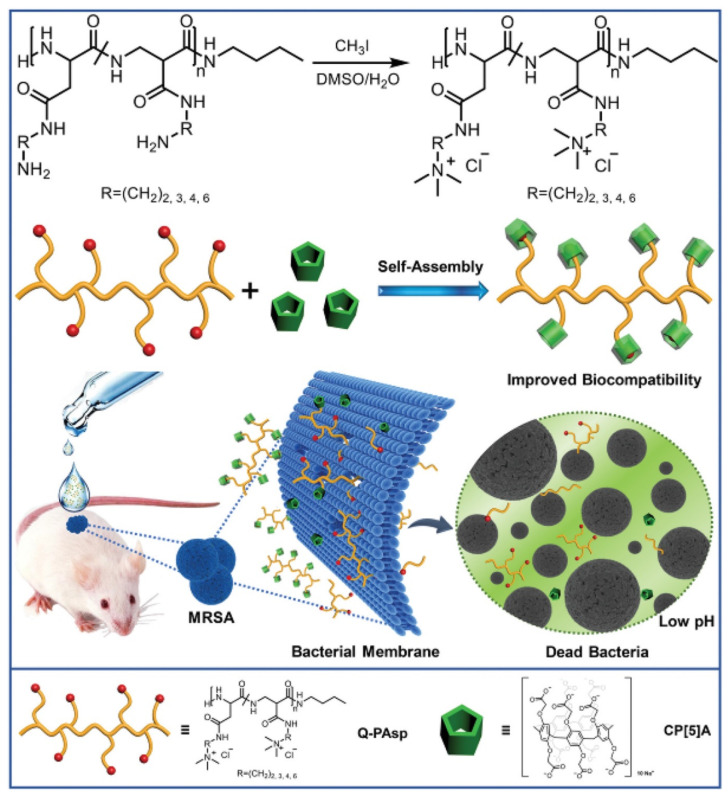
Schematic illustration for construction of supramolecular antimicrobial system and its antibacterial property for healing MRSA-infected wound. Reprinted with permission from Ref. [78]. Copyright 2019 Wiley-VCH.

Yang et al. exploited the supramolecular self-assemblies of cationic polyaspartamides and anionic carboxylatopillar[5]arene (CP[5]A) to target Gram-positive bacteria and mitigate antimicrobial resistance (Figure 6) [78]. The cationic polyaspartamides derivatives were functionalized with quaternary ammonium on the side chains, which contribute to the insertion of the catiomers into the negatively charged bacterial membranes and promotion of membranolysis and cell death. The anionic carboxylatopillar[5]arene (CP[5]A) host was further introduced to complex with the catiomers, which increased the biocompatibility of catiomers. The host–guest interaction endowed the systems with pH-responsiveness, as CP[5]A could depart from cationic quaternary ammonium compounds under acid conditions. Therefore, the biocomposite could selectively eliminate Gram-positive bacteria. In vivo MRSA-infected wound treatment further demonstrated that the system possessed potential values in practical application.

Chan-Park and coworkers also developed block copolymer nanoparticles to remove biofilms of drug-resistant Gram-positive bacteria using nanoscale bacterial debridement [79]. The block copolymer DA95B5, dextran-block–poly((3-5 acrylamidopropyl) trimethylammonium chloride (AMPTMA)-*co*-butyl methacrylate (BMA)), could self-assemble into core–shell nanoparticles with a non-fouling dextran shell and a cationic core. The nanoparticles could diffuse through Gram-positive biofilm and attach to bacterial surfaces. The dextran corona enhanced the bacterial/nanoparticle interactions to promote bacterial detachment from the biofilm, which reduced biofilm biomass. The bacterial debridement mechanism is orthogonal to antibiotic resistance so that it removes biofilms of drug-resistant strains as effectively as those of drug-sensitive strains.

## 5. Supramolecular Self-Assembly for Improvement of aPDT

Antibacterial Photodynamic therapy (aPDT) is a promising way to eliminate bacteria and biofilm infections. In the aPDT process, the reactive oxygen species (ROS) destroy biomolecules in bacterial cells, leading to cell death [80,81,82]. The antibacterial efficiency of aPDT generally is very high, and it also does not induce drug resistance; thus, aPDT has been applied in antibacterial therapy for body infections. However, more excellent aPDT systems are still needed to overcome the drawbacks of aPDT. For instance, the ROS has limited diffusion distances and short lifetimes, and it is inefficient for Gram-negative bacteria due to its complicated cell membrane. The light employed for activation of photosensitizer always shows weak tissue penetration, and the dependence of oxygen for generating ROS also restricts their therapeutic effect. In addition, the reactivity of ROS is very nonspecific, which causes by-effect and lowers their antibacterial performance [83,84,85,86,87]. Therefore, the supramolecular self-assembly strategy has been employed to improve the therapeutic performance of aPDT in two ways, including enhancing the generation of ROS and regulating the interaction between aPDT systems and bacteria.

**Figure 7:**
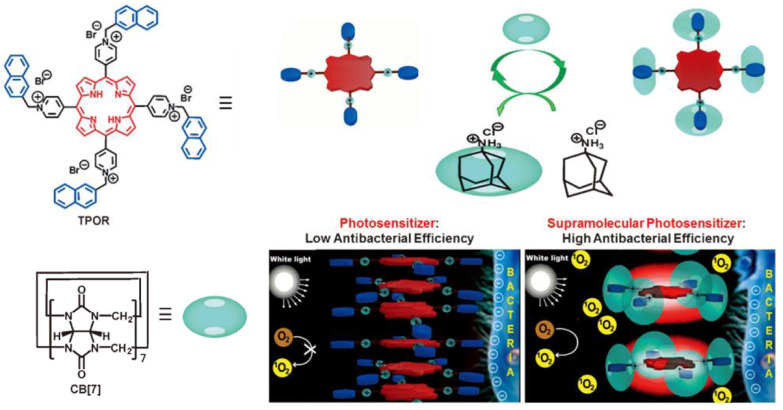
The construction of TPOR/(CB[7])_4_ supramolecular photosensitizers and the mechanism for the enhanced antibacterial efficiency of TPOR/(CB[7])_4_ compared with that of TPOR. Reprinted with permission from Ref. [88]. Copyright 2019 Wiley-VCH.

Due to a strong tendency of π-π stacking in an aqueous solution, the singlet states of photosensitizer are quenched, leading to low ROS generation efficiency. Host–guest complexation of photosensitizer by macrocycles is an efficient way to enhance ROS generation by preventing aggregation of the hydrophobic photosensitizer. For instance, Zhang and coworkers exploited the host–guest complexation of cucurbit[7]uril (CB[7]) and porphyrin derivatives (TPOR) to enhance the antibacterial efficiency [88]. As the naphthalene-methylpyridinium moiety on TPOR can form stable host–guest complexation with CB[7], the supramolecular TPOR/(CB[7])4 assembly was obtained when mixing TORP and CB[7]. The TPOR/(CB[7]4 assembly showed different self-assembling behavior, comparing the self-assembly of TPOR (Figure 7). The TPOR showed more intense fluorescence due to suppression of self-quenching of the excited state, and the ^1^O_2_ generation was also enhanced. Thus, the bacterial inhibition ability was efficiently enhanced by promoting ROS generation. Furthermore, metal ions, including Zn(II) and Pd(II), were introduced into the porphyrin core to enhance its ability to produce ROS and improve the efficiency of PDT [89].

**Figure 8:**
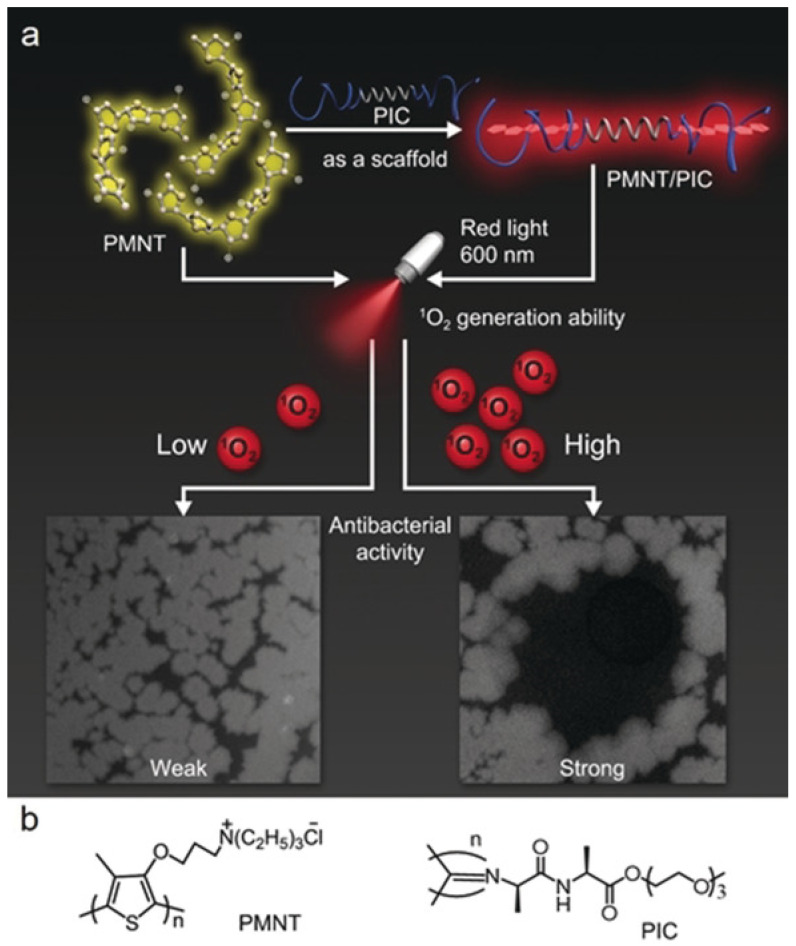
(**a**) Schematic of the assembly of PMNT/PIC, and its antibacterial activity under red light. (**b**) Chemical structure of PMNT and PIC. Reprinted with permission from Ref. [90]. Copyright 2021 Wiley-VCH.

Supramolecular assembly strategy is applied to manipulate the absorbance wavelength of conjugated polythiophene to enhance its aPDT effect under red light irradiation by Xing and coworkers. They fabricated hybrid hydrogels by co-assembling of tri(ethylene glycol)-functionalized polyisocyanide (PIC) and conformation-sensitive conjugated polythiophene, poly(3-(3′-*N*,*N*,*N*-triethylammonium-1′-propyloxy)-4-methyl-2,5-thiophene chloride) (PMNT) (Figure 8). The PIC polymer could serve as a scaffold to trap and align the PMNT backbone into a highly ordered conformation, leading to the generation of a red absorption band. Under red light irradiation, the PMNT/PIC co-assembly showed much higher ROS production than PMNT in its random conformation, leading to efficient aPDT towards various pathogens [90].

**Figure 9:**
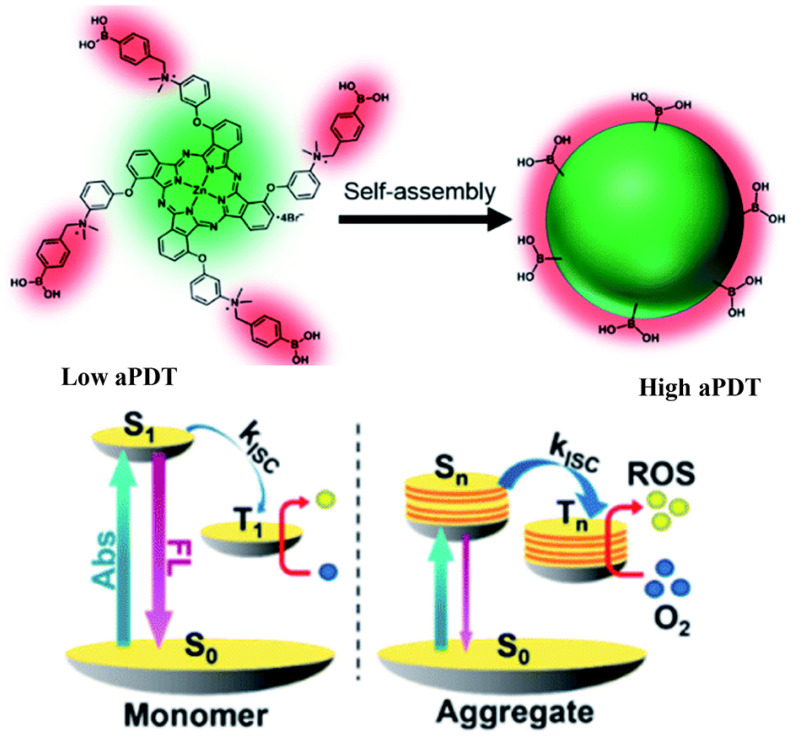
Aggregation-enhanced ROS production of PcN4-BA for combating antibiotic-resistant bacteria. Reprinted with permission from Ref. [91]. Copyright 2020 Royal Society of Chemistry.

Designing aggregation-enhanced ROS production systems is also a novel thread for improving the therapeutic performance of aPDT. Yoon and coworkers developed an aggregation-enhanced photodynamic therapy system for combating antibiotic-resistant bacteria [91]. The self-assembly of the 3-{*N*-(4-boronobenzyl)-*N*,*N*-dimethylammonium} phenoxy-substituted zinc(II) phthalocyanine (PcN4-BA) generated the novel photosensitizer, and the boronic acid functionalized on the PCN4-BA endow the self-assembly with bacteria target capacity (Figure 9). The aggregation of PcN4-BA induces large intermolecular interaction, which further increases the density of electronic states and reduces the energy gap between the excited singlet and triplet states. Furthermore, the enhanced energy overlap between excited singlet and triplet states promotes ISC, resulting in the improvement of ROS generation.

Light harvesting from the antenna dye to photosensitizer was also employed to enhance the therapeutic performance of aPDT by Wang and coworkers [92]. The anionic polythiophene (PTP) and cationic porphyrin (TPPN) formed PTP/TPPN complex through electrostatic interactions, and efficient energy transfer from PTP to TPPN occurred upon white light irradiation, which further promoted ROS generation. After the positive charged PTP/TPPN complex was absorbed on negatively charged bacteria membranes through electrostatic interactions, the singlet oxygen effectively killed the bacteria. 

Regulation of the antibacterial activity of biotics was also accomplished by a supramolecular antibiotic switch. Wang and coworkers fabricated the supramolecular antibiotic switch by the host–guest complexation between cucurbit[7]uril (CB[7]) and the cationic pedants of poly(phenylene vinylene) derivative (PPV) [93]. When the host–guest complexation was retained, the supramolecular antibiotic switch showed low ROS generation efficiency, as the encapsulated PPV was prevented from contacting the surrounding oxygen. Further, the encapsulated PPV also could not bind the bacterial membrane, as the cationic pedants were complexed by CB[7]. Upon competitive replacement by amantadine, the antibacterial activity of the PPV was turned on and showed effective aPDT.

Tang and coworkers also regulated the antibacterial photodynamic effect of oligo(phenylene ethynylene) (OPE) through the supramolecular self-assembly of OPE and DNA [94]. DNA-OPE hydrogel was formed through the electrostatic interactions between positively charged OPE and negatively charged DNA. In the gel state, the OPE showed little biocidal activity towards E.coli, as the OPEs are ‘‘adsorbed’’ by DNA via electrostatic interactions in the gel. When the DNA was hydrolyzed by DNase, the released OPE fully exerted its photodynamic effect on the cell.

A supramolecular self-assembly strategy was also applied to improve the biocompatibility and endow the photosensitizer with acid responsiveness. Recently, Zhang and coworkers fabricated an acid-triggered supramolecular porphyrin photosensitizer to combat bacteria and biofilms. The host–guest complexation of carboxylatopillar[5]arene (CP5) and quaternary ammonium-functionalized tetrafluorophenyl porphyrin (TFPP-QA) formed nanoparticles in aqueous solutions, resulting in the supramolecular photosensitizer [95]. The quaternary ammonium compound showed indiscriminate cytotoxicity to both bacterial cells and normal tissue cells, which greatly limited their clinical applications. After host–guest interactions, the biocompatibility of TFPP-QA was significantly improved. As the CP5 possess excellent pH-responsiveness, the TFPP-QA could release from the nanoparticles when they reach the acidic inflammatory tissue.

Zhang and coworkers also constructed a pH-responsive supramolecular antibacterial photosensitizer AgTPyP@P[5] by the host–guest complexation between a water-soluble photosensitizer silver tetra(*N*-methyl-4-pyridyl) porphyrin (AgTPyP) and carboxylatopillar[5]arene (P[5]) [96]. The host–guest complexation could further self-assemble to form nanoparticles. The introduction of P[5] could reduce the toxicity of AgTPyP to normal tissues. In the acidic bacteria-infected inflammation tissue microenvironment, the host–guest complexation was dissociated and released the AgTPyP. Upon light irradiation, the ROS produced by AgTPyP could effectively kill bacteria.

**Figure 10:**
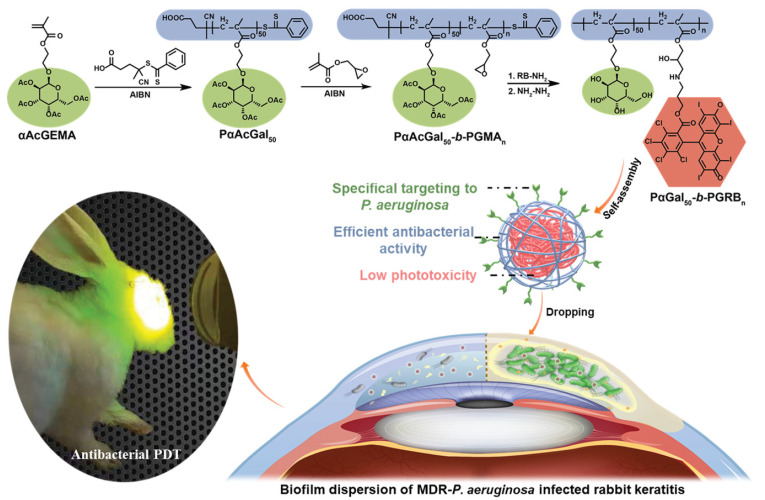
Schematic illustration of MDR bacterial-targeted nanoassembly and its bioapplication in MDR-*P. aeruginosa* biofilm infected rabbit keratitis model. Reprinted with permission from Ref. [97]. Copyright 2021 Wiley-VCH.

Regulating the interaction between aPDT systems and bacteria through supramolecular self-assembly is also a promising way to enhance the therapeutic performance of aPDT and reduce side effects. Xu and coworkers exploited the self-assembly of amphiphilic polymer containing bacteria-target moieties and photosensitizers to combat multi-drug-resistant *Pseudomonas aeruginosa* (MDR-*P. aeruginosa*, Figure 10) [97]. The α-D-galactose on the surface of the nano-assembly targeted the *P*. *aeruginosa* through carbohydrate–protein interaction, and the bacteria were efficiently killed by the generated singlet oxygen from the photosensitizer Rose Bengal.

Multicharged supramolecular aPDT agent was fabricated by the host−guest complexation between hexa-adamantane-appended ruthenium polypyridyl (Ru2) and polycationic cyclodextrin (CD-QAS) to enhance the specific intercalation between negatively charged bacteria membrane and the aPDT agent [98].

## 6. Supramolecular Self-Assembly for Improvement of PTT

Photothermal therapy (PTT) is an antibacterial method dependent on the light-induced heat from the photothermal agents. The locally increased temperature (≥41 ℃) inactivates the microbial cell by inducing protein denaturation and aggregation, disruption of cell membrane integrity, and DNA cross-linking [99,100,101]. Thus, PTT exhibits no drug resistance and holds great potential to treat drug resistance. However, the shortcomings of PTT still need to be overcome. For instance, the light employed for photothermal agents always shows weak tissue penetration. It also always possesses side effects on normal tissues and low efficiencies [102,103]. In order to overcome these shortcomings, a supramolecular self-assembly strategy has been employed to improve the therapeutic performance of PTT in two ways, including improving the photothermal conversion efficiency and regulating the interaction between PTT systems and bacteria.

**Figure 11:**
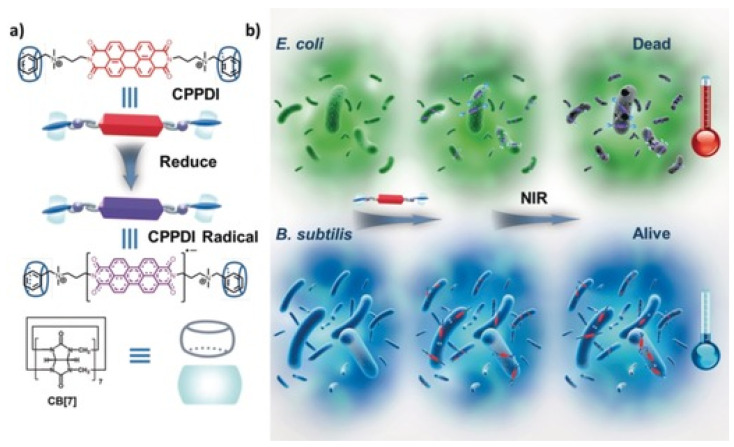
(**a**) Chemical structures of the supramolecular complex (CPPDI) and CPPDI radical anions. (**b**) Diagram of photothermal therapy for supramolecular complex with high selectivity towards *E. coli* over *B. subtilis*. Reprinted with permission from Ref. [104]. Copyright 2017 Wiley-VCH.

Supramolecular radical anions were fabricated to serve as an in situ generated antibacterial near-infrared photothermal agents. The host–guest complexation of PPDI and CB[7] was firstly constructed (Figure 11), which could eliminate nonspecific antibacterial behavior of PPDI thorough preventing the benzyl groups of PPDI from inserting into the membrane of bacteria [104]. Moreover, the host–guest complexation also could promote the stability of PPDI radical anions by preventing them from dimerizing and quenching. When the host–guest complexation was incubated with bacteria with enough reductive ability (*E. coli*), the supramolecular radical anions were in situ generated, which further generated hyperthermia to induce bacterial cell death under near-infrared light irradiation.

In order to improve the therapeutic performance and selectivity of PPT, *E. coli* induced in situ supramolecular polymer was constructed by Xu and coworkers [105]. A bifunctional monomer containing two viologen moieties as end groups and a rigid and positively charged 1,4-diazabicyclo[2.2.2]octane unit as the linker (VDV) was designed to fabricate supramolecular polymer with CB[8]. When incubated with E. coli, the CB[8] and VDV in situ formed a supramolecular polymer integrated with supramolecular dimers of viologen cation radicals on the surface of bacteria, as the viologen moieties were reduced to viologen cation radicals by *E. coli*. The photothermal therapeutic performance of supramolecular dimer of viologen cation radicals was improved by the local enrichment effect of supramolecular polymers and their adsorption onto the bacteria surface. As the viologen moieties were only able to be reduced by *E. coli*, the supramolecular polymer showed high selective photothermal therapeutic performance toward *E. coli*.

**Figure 12:**
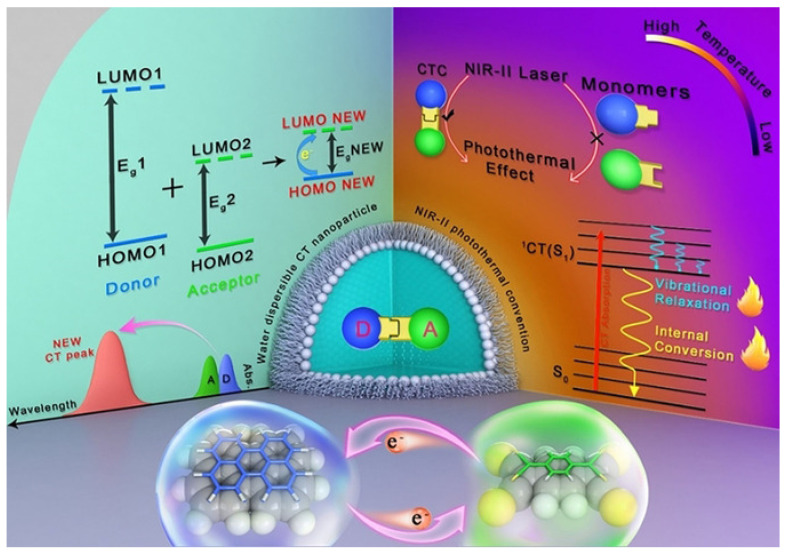
Diagram showing photothermal effect in charge transfer cocrystal nanoparticle. Reprinted with permission from Ref. [106]. Copyright 2021 Wiley-VCH.

A NIR-II photothermal antibacterial agent with much deeper tissue penetration and higher maximum permissible exposure was further fabricated by Lee and coworkers using a charge–transfer complex (Figure 12) [106]. The charge–transfer complex was made up of the donor perylene (PER) and acceptor tetracyanoquinodimethane (TCNQ). The PER with high π-electron donating ability may delocalize electron of TCNA, which would narrow the energy gap of charge–transfer complex. The small energy gap would extend the light-absorbing ability in the NIR-II region and promote a high rate of non-radiative transition. The charge–transfer complex showed an excellent PTT effect on *E. coli* and *S. aureus* under NIR-II laser irradiation.

Regulating the interaction between PTT agents and bacteria through supramolecular self-assembly is an effective way to enhance the therapeutic performance of PTT. A supramolecular carbohydrate-functionalized two-dimensional (2D) surface was fabricated by Haag and coworkers to selectively capture *E. coli* and enhance the PTT of the graphene. The supramolecular carbohydrate-functionalized two-dimensional (2D) surface was constructed by host–guest complexation between hepta mannosylated β-CD (ManCD) and the adamant group on thermally reduced graphene oxide. The host–guest complexation on the surface of thermally reduced graphene oxide not only increased the intrinsic water solubility but also endowed the surface with effective *E. coli* capture capacity through multivalent interactions. Upon IR laser irradiation, the captured *E. coli* was killed effectively by the photo-induced heat from the graphene oxide [107].

## 7. Supramolecular Self-Assembly for the Improvement Phagocytosis of Macrophage

**Figure 13:**
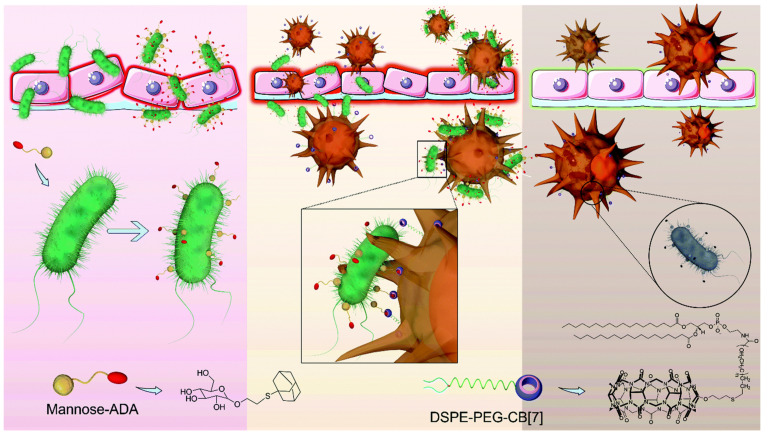
Enhanced antibacterial function of a supramolecular artificial receptor-modified macrophage. Reprinted with permission from Ref. [108]. Copyright 2022 Royal Society of Chemistry.

The phagocytosis of bacteria by macrophages acts as the front line to protect the human body from bacterial pathogens. The pathogens’ clearance efficiency is always low due to the relatively slow encounter and insufficient capture of bacteria by macrophages. Wang and coworkers developed a supramolecular artificial receptor-modified macrophage (SAR-Macrophage) to enhance the recognition and capture of bacteria in the systemic circulation. SAR-Macrophage was fabricated by decorating CB[7] on the macrophage surface via inserting DSPE-PEG-CB[7] (1,2-distearoyl-sn-glycero-3-phosphoethanolamine-poly(ethylene glycol)-CB[7]) into the surface membrane of the cells (Figure 13). *E. coli* was decorating with adamantyl (ADA) groups by the mannose-ADA via specific mannose–FimH interactions. Based on the strong and multipoint host–guest interactions between CB[7] on SAR-Macrophage and ADA on the *E. coli*, the SAR-Macrophage could significantly recognize *E. coli* and catch and internalize more pathogens. The promoted recognition and capture of *E. coli* induced the M1 polarization of macrophages to generate ROS and effectively killed the intracellular bacteria. This work provides a supramolecular cell-engineering approach for potential antibacterial applications [108].

## 8. Summary

Antibiotic resistance has caught the attention of researchers from different fields, and plentiful multidisciplinary studies for combating antibiotic resistance have been reported in recent years. Supramolecular assembly is tunable, modular, and responsive, as the formation of assembly is based on noncovalent interactions. Supramolecular self-assembly provides a promising and versatile strategy to construct new antibacterial agents to combat antibiotic-resistant infections. In this review, we hope we have shown the recent development of antibacterial agents based on the supramolecular self-assembly strategies, which include the supramolecular self-assembly of antibiotics, antibacterial peptides, cationic surfactants and polymers, antibacterial photodynamic therapy agents, antibacterial photothermal therapy agents, as well as supramolecular assembly strategy engineered macrophage-bacteria interaction for promoting phagocytosis. These new antibacterial agents showed enhanced antibacterial activity, or the intrinsic shortcomings of the motifs were improved after supramolecular self-assembly.

The current state of the art for antibacterial agents based on supramolecular self-assembly strategy exhibits strong potential to treat drug-resistant bacterial infections in the near future. However, there are still some issues that require to be added to promote the clinical translation of the novel antibacterial agents. First, the new antibacterial agents need to possess excellent biocompatibility. Biocompatibility is a basic requirement for constructing the new antibacterial agents, and the supramolecular self-assembly strategy also could be considered for the cytotoxicity of the potential reported antibacterial agents. Second, the stability of the new antibacterial agents needs to be systematically investigated. The new antibacterial agents exert their enhanced activity through the positive effects induced by supramolecular self-assembly, but the supramolecular self-assembly may show instability in the complex biological media. The revelation of the mechanism of the new antibacterial in the complex biological media will help in developing effective strategies for future applications. Third, the generation of resistance needs to be carefully evaluated during the research stage. As the resistance could be developed rapidly, the evaluation of resistance generation promotes feasibility for clinical tests. Last but not least, more new antibacterial agents based on supramolecular self-assembly need to be fabricated.

## Data Availability

Data sharing not applicable.
